# Optimizing the xWORM assay for monitoring hookworm larvae motility

**DOI:** 10.3389/fpara.2023.1189872

**Published:** 2023-06-19

**Authors:** Danica Lennox-Bulow, Luke Becker, Alex Loukas, Jamie Seymour, Michael Smout

**Affiliations:** ^1^ Tropical Australian Stinger Research Unit, James Cook University, Cairns, QL, Australia; ^2^ Australian Institute of Tropical Health and Medicine (AITHM), James Cook University, Cairns, QL, Australia

**Keywords:** xWORM, drug screening and discovery, hookworm larvae, helminth culture, assay conditions, method optimization

## Abstract

Parasitic worms (helminths) infect almost all taxa across the animal kingdom, and pose significant challenges to public health and economies, particularly in developing countries. To address this problem, researchers have developed various tools to measure the motility and viability of helminths. However, the conditions used in anthelmintic screening assays are often not optimized, and can vary considerably between research teams. These unoptimized conditions may impact novel drug screens, as little is known about the effects of different conditions on the health of the target parasites. To improve future research, this study determined the effects of key assay parameters including, media type, media concentration, in-well parasite density, and assay duration on the infective third-stage larva (L3) of two types of hookworms, namely *Nippostrongylus brasiliensis* in rodents, and *Necator americanus* in humans. Conditions were screened over several days using the xCELLigence worm real-time motility assay (xWORM); a real-time impedance-based helminth motility assay using the xCELLigence system with 96-well microplates. While results varied depending on the species and media used, the study found that 500–1,000 L3/200-µL and a media concentration of 3.13–25% generally produced good to excellent assay conditions. The findings of this study can guide the future selection of xWORM assay parameters for novel drug trials involving these parasite species and serve as a suggested model for optimizing trial conditions for alternative parasite targets and assays.

## 1 Introduction

One of the major drawbacks of conducting an experiment on any living organism outside of its natural habitat is the potential for the artificial conditions to confound results (Morgan and Tromborg, 2007; Akhtar, 2015). As such, selecting the abiotic factors that make up the artificial conditions requires careful consideration of the ecology and biology of the target organism, whilst, in some cases, balancing the requirements of the equipment being used [i.e., the xCELLigence^®^ Real Time Cell Analyzer (RTCA; ACEA Biosciences) system]. This becomes particularly important when the experimental design involves measuring a physiological response, (i.e., parasite motility) to a treatment, such as novel drug targets with a predetermined or desired effect. Recently, a number of screening techniques with reduced subjectivity and higher-throughput capabilities have emerged to measure the physiological responses (i.e., motility) of parasitic worms to novel drug targets, including; video assays (Paveley et al., 2012), enzymatic assays (Mansour and Bickle, 2010), colorimetric assays (Tritten et al., 2012), fluorescence (Peak et al., 2010), and other mechanisms (Howe et al., 2015; Lalli et al., 2015). Among these was the development of the xCELLigence worm real-time motility (xWORM) assay, (Smout et al., 2010; Rinaldi et al., 2015).

The xWORM assay uses the xCELLigence real-time cell analyzer system, which is principally used to monitor cellular events in real time, to measure parasite motility through fluctuations in electrical impedance (Smout et al., 2010). To measure electrical impedance, the xCELLigence system applies an electrical potential across gold interdigitated microelectrodes fused to the bottom surface of each well of a 96-well E-plate. When cells or helminths make contact with these electrodes, it increases the resistance inside the well, which is relayed to the analyzing software as an increase in the cell index (CI) value. Conversely, where there is no contact, there is less resistance, resulting in a decrease in the CI value. As described by Smout et al. (2010), live parasites writhe in culture, as they do *in vivo*, and constantly vary their contact with the electrodes on the bottom surface of the E-plate. This induces a variable resistance that results in an irregular CI value with an amplitude that is proportional to parasite motility. When the helminths become weakened or die, the resistance across the electrodes stabilizes as the helminths are stationary on the base of the E-plate. Since its conception, the xWORM assay has had numerous applications across helminth species and life stages, including screening novel compounds for anthelmintic activity, the determination of the 50% inhibitory concentration of numerous pre-existing anthelmintic treatment options, and also the detection of phenotypic resistance to gold standard anthelmintics (Smout et al., 2010; Silbereisen et al., 2011; Tritten et al., 2012; Rinaldi et al., 2015; Wangchuk et al., 2016).

Interestingly, some of the baseline assay conditions that the parasites are exposed to, i.e., media type, media concentration, and in-well parasite density, vary considerably between published works, including those by Smout et al. (2010); Tritten et al. (2012), and Rinaldi et al. (2015). This is particularly true for assays involving the larval stages of helminths [i.e., infective second- to fourth-stage larvae (L2–L4)]. In some cases, these conditions have been shown to underpin the success of the assay (Tritten et al., 2012). However, to date, very little is known about their effects on both the measurement system and the target parasites. Therefore, the aim of this study was to measure the effect of key assay parameters, including, media type [phosphate-buffered saline (PBS) and Dulbecco’s modified Eagle’s medium (DMEM)], media concentration (3.13–100%), and in-well parasite density (67–2000 L3/200-µL well volume) on the motility of infective third-stage (L3) hookworm larvae, specifically *Nippostrongylus brasiliensis* (infective rodent hookworm larvae) and *Necator americanus* (infective human hookworm larvae). The goal of this was to identify and select the combination of xWORM assay parameters suitable for future studies aiming to screen novel compounds for anthelmintic activity against these species.

## 2 Materials and methods

### 2.1 *N. brasiliensis* cultivation

The cultivation of *N. brasiliensis* L3 followed a protocol outlined by Camberis et al. (2003). Using a 21-gauge needle, 250-µL of 1 × PBS solution containing ≈ 2500 – 3000 *N. brasiliensis* L3 was injected subcutaneously between the shoulder blades of female Sprague Dawley^®^ rats (ethics approval number A2626). Rats were monitored daily for physical and/or behavioral abnormalities. Rats were transferred to a new cage on the fifth day post infection. Infected fecal pellets were collected on days six and seven post infection. To culture the parasite, feces were first hydrated in reverse-osmosis (R/O) H_2_O and mixed thoroughly to form a smooth slurry. Granulated and fine particulate activated charcoal was then added to the slurry at a ratio of 2 : 1 or until the mixture resembled natural soil. The charcoal mixture was distributed onto R/O H_2_O dampened filter paper (80-mm rounded), which had been placed within 90-mm sterile plastic Petri dishes. Plates were then set to incubate in the dark at 26°C. The larvae were left undisturbed for five days to allow for migration to the periphery of the plate. Collecting *N. brasiliensis* L3 involved gently washing the parasites off the sides and lid of the plate with a Pasteur pipette and R/O H_2_O. Collected *N. brasiliensis* L3 were transferred to a 50-mL Falcon™ tube. To calculate the number of *N. brasiliensis* L3 harvested, the Falcon tube was topped up with R/O H_2_O to the nearest 10-mL gradation. Subsequently, 100-µL of the original solution was aliquoted into a 1.5-mL Eppendorf tube containing 900-µL of R/O H_2_O. The number of *N. brasiliensis* L3 in a 100-µL subsample of the diluted solution was then counted using a top light microscope and extrapolated to yield the total number of parasite larvae harvested.

### 2.2 *N. americanus* cultivation

Fecal material for culture was obtained from a chronically infected donor from James Cook University who was carrying an *N. americanus* strain originating from Madang, Papua New Guinea. The donor was a 50-year-old male who had been inoculated with 20 *N. americanus* L3 in May 2020, with human research and ethics approvals provided by James Cook University (ethics approval number H5936). The donor was confirmed to be negative for transmissible blood-borne viruses [i.e., human immunodeficiency virus (HIV), hepatitis B virus (HBV), and hepatitis C virus (HCV)] and for infection with significant bacterial enteropathogens (*Salmonella, Shigella*, and *Campylobacter*). Fecal cultures were established within 48 hours of defecation following the procedure outlined in section 2.1.

### 2.3 Measuring parasite motility using xCELLigence RTCA

In previous studies, the xCELLigence RTCA has been used to differentiate between live and dead helminths across several species and developmental stages (Smout et al., 2010; Silbereisen et al., 2011; Tritten et al., 2012; You et al., 2013; Zeraik et al., 2014). Although this was chiefly used to assess the efficacy of novel anthelmintics, our aim was to measure the effect of baseline assay parameters, i.e., media type, media concentration, and in-well parasite density, on the motility of *N. brasiliensis* L3 (rodent hookworm) and *N. americanus* L3 (human hookworm) for the purpose of optimizing xCELLigence measuring conditions for future novel drug screening trials (**Supplementary Data 1**).

To achieve this, we used an adjusted version of the xWORM assay developed by Rinaldi et al. (2015). In brief, PBS and DMEM media were prepared at six specified concentrations: 100%, 50%, 25%, 12.5%, 6.25%, and 3.13%. These concentrations were achieved by diluting the media with deionized (Milli-Q^®^) H_2_O. Subsequently, 150-µL of the prepared media and 2% antibiotic–antimycotic (AA) were added to each well of a 96-well E-plate. Plates were placed into an xCELLigence RTCA single-plate (SP) instrument to undergo an initial background read. Live (i.e., treatment group) and heat-killed (i.e., positive control group) *N. brasiliensis* or *N. americanus* L3 at densities of 2000, 1000, 500, 250, 125, and 67 L3/200-µL well volume were then added to all wells in triplicate, apart from three that were reserved as a media-only control group. Parasites were added in 50-µL aliquots of solution made up with chosen media and 2% AA. Media-only control wells were devoid of parasites and consisted of 200-µL of the chosen media and 2% AA. As hookworms are less active in an environment lacking the presence of a mammalian host, plates and RTCA SP instruments were incubated at 37°C in a humidified atmosphere containing 5% CO_2_ throughout the duration of the experiment to simulate mammalian body temperature, and thus stimulate parasite motility. Parasite motility was registered at 25 kHz in regular user-defined reads at 15 second intervals over 72 hours. Parasite motility was measured for all media type, media concentration, and in-well parasite density combinations for both *N. brasiliensis* and *N. americanus* L3. CI figures were produced in GraphPad Prism 9 (GraphPad Software Inc., CA, USA).

Given that CI represents the real-time electrical impedance within all wells of a 96-well E-plate, CI scatter curves are a great way to screen for data corruptions (i.e., faulty wells) prior to proceeding with further data conversions and analyses. However, although changes to parasite motility can be roughly identified in the raw CI scatter, with multiple wells and conditions, comparisons can become difficult. Therefore, as a more intuitive way to visualize and compare parasite motility, CI data were converted to a motility index (MI).

### 2.4 MI transformation

MI is a measure of the amplitude of the CI curve scatter over time. As described by Smout et al. (2010), MI is calculated as the standard deviation over 800 data points of the CI difference from the rolling average over 30 data points. To calculate the MI, raw CI readings from the RTCA Software Pro were exported to Microsoft Excel^®^ (Microsoft Corporation, Redmond, WA, USA). To account for background noise, MI data was blank adjusted by subtracting each data point for treatment and positive control groups from the corresponding data point of the media-only control. Hypermotility was observed in all MI traces over the first 24 hours of the assay, and thus was designated as an equilibration period and excluded from further analyses. As such, the 24 hours after assay commencement will hereafter be referred to as treatment time 0.

In contrast to CI scatter plots, MI curves are an intuitive way of visualizing parasite motility over time (**Supplementary Data 2**). Another benefit of this conversion is that MI is suitable for use in a wide range of statistical analyses, the most common for drug trials being dose response. In this case, MI data are subsequently converted to a percentage of maximum motility, which can be used to generate dose–response curves for traditional IC_50_ calculations. However, MI can also be used in a wide range of hit selection models, such as strictly standardized mean difference prime (SSMD’). Therefore, to determine the optimal media type, media concentration, and in-well parasite density for xWORM assays with *N. brasiliensis* and *N. americanus* L3 we used SSMD’.

### 2.5 Assessing assay quality using SSMD’

SSMD’ is a hit selection model developed by Zhang (2011). SSMD’ is a way of measuring the size of an effect from the control group, relative to its variation, and is particularly useful for comparisons across groups that measure the same effect but operate under different physical parameters. For xWORM data, SSMD’ scores represent the mean difference in parasite motility between treatment groups, and corresponding heat-killed parasite controls, relative to the variation within each group. Hence, treatment groups with the greatest magnitude of difference from the heat-killed parasite control group coupled with low variability between replicates, yield high SSMD’ scores. Previous studies have used SSMD’ to determine the optimal measurement frequency, and in-well density for *Schistosoma mansoni* egg hatching and cercariae motility (Rinaldi et al., 2015). For future xWORM trials aimed at screening novel compounds for anthelmintic activity against *N. brasiliensis* and *N. americanus* L3, we targeted conditions that yielded a stable motility with the greatest magnitude of difference from the positive control group, and low variance between replicates. To identify these conditions quantitatively, SSMD’ values were generated for each condition combination and parasite species using the following protocol:

First, raw MI data were blank adjusted by subtracting all data points from the average MI of the corresponding media-only control to account for background signal. The standard deviation of each treatment and heat-killed parasites control group was then calculated across the triplicates for each time point. SSMD’ scores were then calculated for each hookworm species (i.e., *N. brasiliensis* L3 or *N. americanus* L3) and assay condition combination (i.e., media type, media concentration, and in-well parasite density) from treatment time 0 (i.e., 24 hours after assay commencement) up to 48 hours as:


(1) $$SSMD^{\prime }\;=\;\left|\frac{\bar{AC}\;-\;\bar{HK}}{\sqrt{s_{AC}^{2}\;+\;s_{HK}^{2}}}\right|.$$

$\bar{AC}$ is the average MI value of hookworm larvae in *y* “assay condition” (i.e., media type, media concentration, and in-well parasite density combination). $\bar{HK}$ is the average MI value of “heat-killed” hookworm larvae. $\;s_{AC}$ is the standard deviation of the MI of hookworm larvae in *y* “assay condition”. $\;s_{HK}$ is the standard deviation of the MI of ‘heat-killed’ hookworm larvae.

SSMD’ is calculated as the mean of the differences divided by the standard deviation of the differences between the treatment group (parasites in various conditions) and a negative reference (heat-killed parasites in the same conditions), where the selected assay conditions (i.e., the media type, media concentration, and in-well parasite density combination) and their matching positive control (i.e., heat-killed parasites) have mean values designated as $\bar{AC}\;$ and $\bar{HK}$ with standard deviations of $s_{AC}\;$ and $s_{HK}$ , respectively.

Identifying the optimal conditions (i.e., hit selection) for xWORM assays with *N. brasiliensis* and *N. americanus* L3 was based on the classification system outlined by Zhang (2011, **Table 1**), whereby assay conditions (i.e., media type, media concentration, and parasite in-well density combinations) with scores between zero and three, and between three and five, were considered to be poor and inferior, respectively. However, assay conditions that scored between five and seven were considered good, and those scoring greater than seven were considered excellent.

**Table 1:** Classification of assay quality using SSMD’ values based on Zhang (2011).

Assay quality	Absolute SSMD’
**Excellent**	> 7
**Good**	5–7
**Inferior**	3–5
**Poor**	0–3

SSMD’, strictly standardized mean difference prime.

Isopleths were generated in MATLAB^®^ (MathWorks R2022a) and used to visualize the interaction between parasite motility (as determined by the xWORM assay) and baseline assay condition combinations on mean SSMD’ scores. Optimal condition ranges were further compared between parasite species (*N. brasiliensis* and *N. americanus* L3) and across two time points (24- and 48-hours treatment time). Conditions that produced highly variable MI traces were deemed as outliers. The mean SSMD’ values for the outliers were replaced with the average SSMD’ of the adjacent scores.

## 3 Results

### 3.1 SSMD’ assay quality assessment for *N. brasiliensis* L3

**Figure 1:**
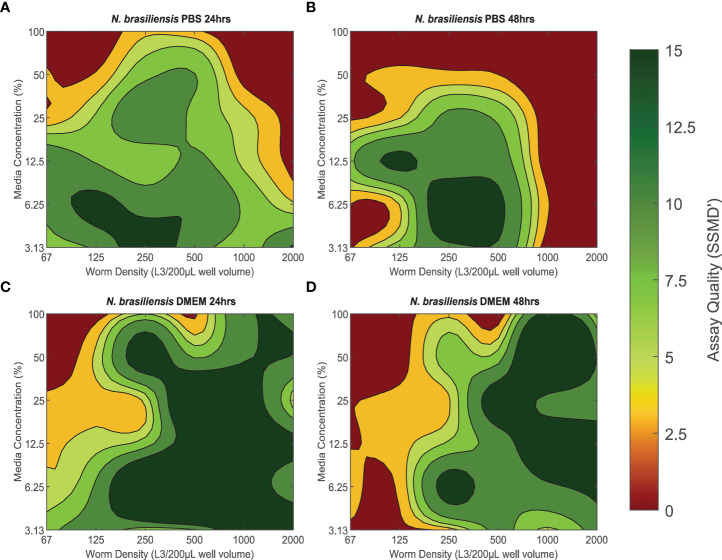
**(A–D)** The effect of baseline conditions on *Nippostrongylus brasiliensis* L3 xWORM [xCELLigence Worm Real-Time Motility] assay quality over time. SSMD' [Strictly standardized mean difference prime] was used to assess xWORM assay quality for the infective larval (L3) stage of the rodent hookworm (*N. brasiliensis*). SSMD’ scores were calculated for each baseline condition combination of media type PBS [Phosphate-Buffered Saline] or DMEM [Dulbeccos Modified Eagle's Medium], media concentration (3.13–100%), and in-well parasite density (67–2000 L3/200 µL well volume) at two time points (24 and 48 hours). Isopleths are defined as the mean SSMD’ score, where a score of < 3 indicates a poor assay; 3–5, an inferior assay; 5–7, a good assay; and > 7, an excellent assay.

The quality of xWORM assays with *N. brasiliensis* L3 were affected by both media concentration and parasite density (**Figure 1**). These trends also varied between media types (PBS/DMEM) and over time (24- and 48-hour treatment time points). PBS conditions that produced good to excellent assay scores over 24 hours ranged between ≈ 3.13–25% media concentration with 67–1000 L3/200-µL well volume (**Figure 1A**). This range was slightly more restricted when measuring over 48 hours, where ≈ 3.13–25% media concentration and 125–800 L3/200-µL well volume yielded good to excellent assay scores (**Figure 1B**). However, PBS conditions that had the highest assay quality (> 7, excellent assay) over time were with a media concentration of ≈ 3.13–6.25%, coupled with a parasite density of 250–500 L3/200 µL well volume. DMEM conditions that produced good to excellent assay scores over 24 hours had a slightly broader range inclusive of 3.13–100% media concentrations and parasite densities between 250-2000 L3/200-µL well volume (**Figure 1C**). These conditions maintained mostly good to excellent assay scores over 48 hours, although some exhibited a slight drop in quality over time (**Figure 1D**). The DMEM conditions that maintained the strongest assay scores (> 7) between the 24- and 48-hour time points were 50–100% media with 1000–1500 L3/200-µL well volume, 12.5–25% media with 500–1000 L3/200-µL well volume, and 6.25–12.5% media with 1000-2000 L3/200-µL well volume.

### 3.2 SSMD’ assay quality assessment for *N. americanus* L3

**Figure 2:**
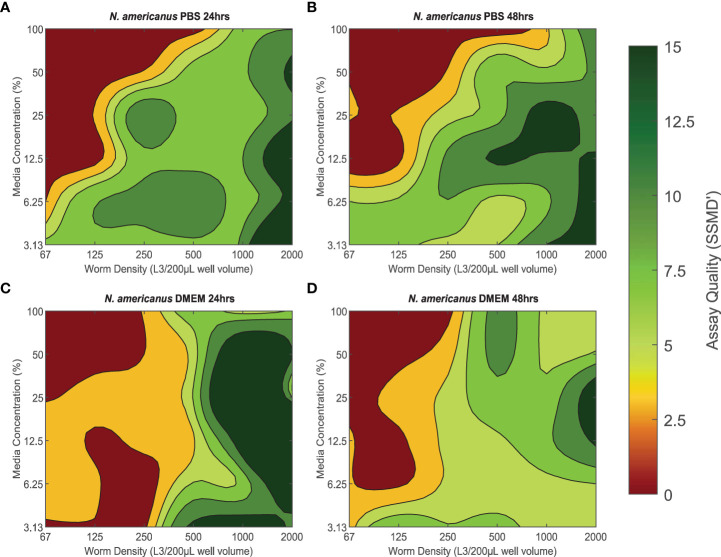
**(A–D)** The effect of baseline conditions on *Necator americanus* L3 xWORM [xCELLigence Worm Real-Time Motility] assay assay quality over time. SSMD' [Strictly standardized mean difference prime] was used to assess xWORM assay quality for the infective larval (L3) stage of the human hookworm (*N. americanus*). SSMD’ scores were calculated for each baseline condition combination of media type (PBS [Phosphate-Buffered Saline] or DMEM [Dulbeccos Modified Eagle's Medium]), media concentration (3.13–100%), and in-well parasite density (67–2000 L3/200 µL well volume) at two time points (24 hours and 48 hours). Isopleths are defined as the mean SSMD’ score where < 3 indicates a poor assay; 3–5, an inferior assay; 5–7, a good assay; and > 7, an excellent assay.

The quality of xWORM assays with *N. americanus* L3 were affected by media concentration and parasite density (**Figure 2**). These trends also varied between the two media types as well as over time. PBS conditions yielding good to excellent assay scores over 24 hours were ≈ 3.13–30% media concentration with a parasite density of ≈ 250–2000 L3/200-µL well volume (**Figure 2A**). These conditions maintained mostly high SSMD’ scores, with only slight variance over 48 hours (**Figure 2B**). The PBS conditions with the highest assay quality over time were ≈ 3.13–12.5% media with an in-well parasite density between ≈ 1500–2000 L3/200-µL well volume. DMEM conditions with *N. americanus* L3 exhibited a similar pattern to what was observed for PBS conditions (**Figures 2C, D**). Good to excellent assay scores were recorded for all DMEM media concentrations (3.13–100%) with parasite densities between 500–2000 L3/200-µL well volume over both the 24- and 48-hour time periods. However, assay scores falling within the excellent category were much less abundant at the 48-hour time point than at the 24-hour time point.

## 4 Discussion

The results of this study demonstrate that assay quality is affected by the combination of media type, media concentration, and in-well parasite density selected, as well as assay duration. We further show that the effect these parameters have on assay quality differs between two closely related hookworm species of the same life stage, *N. brasiliensis* and *N. americanus* L3. For instance, *N. americanus* performed better in PBS media over longer time periods than *N. brasiliensis*. However, *N. brasiliensis* performed better overall in DMEM media. Additionally, in PBS media, higher densities of *N. americanus* resulted in a stronger assay in most cases. Conversely, this resulted in a weak assay for *N. brasiliensis*, particularly with high concentrations of PBS media, and assays conducted over longer periods of time.

Since its conception, the xWORM assay has been used to measure the motility of numerous species and life stages of helminths. However, one of the most striking elements within the methodology of the published literature is that selected assay conditions including media type, media concentration, and in-well parasite density, vary considerably. Across several studies, the type of media used in xWORM assays has been selected to mimic the natural environment of the parasite at that life stage (Smout et al., 2010; Rinaldi et al., 2015). For instance, as adult and some later larval stages are known to occur *in vivo*, cell culture medium [i.e., DMEM or Roswell Park Memorial Institute (RPMI)] was selected in attempt to replicate the conditions experienced inside a host animal. Conversely, as eggs and early larval stages commonly occur in the environment, such as in soil or bodies of freshwater, saline solutions (i.e., PBS) are often selected. Nonetheless, the distinct preferences of human and rodent hookworm larvae for DMEM and PBS media suggest that choosing the appropriate medium may be more complex than previously assumed. This study highlights that factors such as media concentration and in-well parasite density should be considered in conjunction with media type for a more comprehensive approach. However, in previous xWORM studies, these conditions have frequently been selected based on subjective choice or constrained by resource availability, rather than systematic evaluation. In some instances resource constraints, possibly combined with an unsuitable pairing of media type and concentration, have resulted in the failure of the assay. According to a study by Tritten et al. (2012), the third-larval stage of the hookworm *Ancylostoma ceylanicum* was immeasurable at densities of 100 L3/well which was presumed to be related to the activity level of the species. On the other hand, previously successful assays, particularly those involving larval life stages, have utilized high numbers of parasites. One study, for example, measured *Haemonchus contortus* L3 at a density of 3000 L3/well, and *Strongyloides ratti* L3 at a density of 300 L3/well (Smout et al., 2010). Similarly, using SSMD’ as an indicator for assay quality, another study determined that 4500 *Schistosoma mansoni* cercariae/well yielded the strongest assay for the selected media (10% PBS) and duration (16–20 hours; Rinaldi et al., 2015). This has resulted in the belief that xWORM assays require large numbers of parasites to be successful (Tritten et al., 2012). However, obtaining high numbers of parasites can be challenging due to the complex life cycle of most parasite species, which involves multiple morphological stages, and sometimes, several host animals (Ahmed, 2014). This can render artificially simulating the parasites natural conditions difficult from both a cultivation and experimental perspective (Ahmed, 2014).

In experimental settings, ensuring suitable conditions for monitoring parasite health *in vitro* becomes increasingly challenging when researchers must simultaneously accommodate the specific requirements of the measuring system in use. For example, to assess parasite motility through changes in electrical impedance using the xCELLigence system, an ionic solution is required in the measuring environment. The ionic strength of the media needed for the system to accurately monitor parasite motility is likely influenced by various ecological and physiological factors related to the study organism, particularly physical size and activity level. However, the tolerance of the study organism to these conditions is also anticipated to differ significantly among parasite species and life stages. Hence, when selecting assay conditions, it is crucial to strike a balance between meeting the system’s requirements and preserving the study organism’s viability to ensure the accuracy and success of the research. It is crucial to recognize that the influence of artificial conditions on parasite behavior and sensitivity, especially in relation to treatments, is currently unknown. Nevertheless, striving to simulate the natural environment of the parasite as closely as possible is likely the most productive set of conditions for xWORM assays.

Previous studies have shown that the sensitivity of impedance measurements captured by xCELLigence can also be improved by modifying the system’s frequency settings (Rinaldi et al., 2015). This proved to be particularly useful when measuring the hatching frequency of *S. mansoni* eggs, which require near-freshwater conditions to maintain their viability (Rinaldi et al., 2015). When this was combined with the appropriate combination of media type, media concentration, and in-well parasite density tailored to the study organism and intended assay duration, we showed that smaller parasite numbers can yield a strong assay. Although the overall strongest assay conditions for both *N. brasiliensis* and *N. americanus* were determined to involve high parasite numbers (> 125 L3/200-µL well volume), as little as 67 L3/200-µL well volume yielded high scores under certain conditions, specifically with a low media concentration, and shorter assay duration. However, although frequency was not considered by this study due to the promising results obtained at 25 MHz (Rinaldi et al., 2015), parasites or specific conditions may benefit from additional frequency optimization. Similarly, while high numbers of *S. mansoni* (4500 cercariae/well) in select conditions reportedly produced the strongest assay, a good assay could still be achieved with 562 cercariae/well (Rinaldi et al., 2015). Our results suggest that adjusting media type and/or concentration may potentially further allow for lower cercariae densities while maintaining a high assay quality.

While this study offers a preliminary framework for optimizing the xWORM assay, the effect of several other critical assay parameters, including pH, temperature, and the availability of nutrients and oxygen, requires further investigation. For instance, our findings illustrated that increased larval densities could sometimes impair assay quality, as evidenced in the case of *N. brasiliensis* L3 in PBS media. A plausible explanation for the observed detrimental effects associated with increased larval densities could be fluctuations in critical media parameters, such as pH, over time. This phenomenon is known to be associated with decreased viability of mammalian cells maintained *in vitro*. As cells generate waste products (for instance, lactate), they can exceed the buffering capacity of the culture media, especially when maintained at high densities and/or over prolonged durations, leading to pH fluctuations (Gaze et al., 2014). Similarly, hookworm adults, including *N. americanus*, excrete and secrete proteinaceous material which that reportedly enables the parasite to avoid detection by the host’s immune system (Hewitson et al., 2009; Logan et al., 2020). Following manual extraction from the host and subsequent *in vitro* cultivation, viable adult hookworms continue to generate excretory and secretory (ES) products and reproductive materials (Logan et al., 2020), which over time likely affects parasite vitality. Although it is currently uncertain, it is plausible that third-stage larvae also generate potentially toxic by-products, the effects of which could be exacerbated by high in-well larval densities.

Conversely, as exemplified by *N. americanus* L3 in both PBS and DMEM media, high parasite densities were sometimes required for preserving parasite vitality. Higher in-well parasite densities may be advantageous when monitoring the motilities of certain species such as *N. americanus*, which could exhibit lower activity levels. In this scenario, higher numbers would be required to amplify motility signals. Alternatively, these species might experience stimulation through body contact. For instance, third-stage larvae are known to detect hosts in nature through positive thigmotropism (Haas et al., 2005; Gaze et al., 2014). Moreover, upon hatching in culture, hookworm larvae rapidly disperse in all directions, moving toward the periphery of the plate (Camberis et al., 2003; Camberis et al., 2016). This behavior could represent an evolutionary adaptation aimed at enhancing the success of transmission to a host. Specifically, close proximity with other larvae might trigger a response in the larvae to move away from each other to expand their spatial footprint, thereby increasing the likelihood of encountering a viable host. However, this is purely speculative and requires a deeper understanding of parasite behavior.

Another factor to consider when selecting xWORM assay conditions is oxygen and nutrient availability. A recent study by Hass et al. (2023) reported that in-well media volume significantly impacts the metabolic activity of retinal pigment epithelium (RPE) cells by affecting oxygen and nutrient availability. Researchers may initially overlook this as a potential factor when measuring the motility of L3, as it is widely believed that they are in a state of arrested development and survive solely on the energy accumulated during previous larval stages (Gaze et al., 2014). However, studies have shown that various environmental stimuli can trigger larval metamorphosis and the resumption of feeding, including warmer temperatures and the presence of serum or host-specific secretions such as sweat (Hawdon and Schad, 1990; Kumar and Pritchard, 1994; Haas et al., 2005; Pasuralertsakul and Ngrenngarmlert, 2006). The incubation temperature commonly used in xWORM trials (37°C) to stimulate worm motility could thus trigger an artificial metamorphosis in hookworm parasites from a free-living to a parasitic lifestyle and/or a resumption of feeding. This could potentially account for the observed decline in assay quality for *N. brasiliensis* L3 in PBS media over a 48-hour period. Therefore, future studies should aim to explore the effect of serum supplementation and oxygen availability on xWORM assay quality, particularly for studies conducted over longer time frames.

It is also worth considering that extended exposure to elevated temperatures may be detrimental to ensheathed hookworm larvae. As the sheath acts as a thermal buffer in a dynamic environment, prolonged heat exposure could trap heat and potentially decrease larval vitality. The rate at which larvae successfully shed their protective sheaths in response to artificially induced environmental stimuli, such as temperature or the presence of serum, is currently unknown and should be a focus of future research. However, this phenomena may explain the broad spectrum of optimal conditions identified in this study, potentially reflecting variations in the ability of third-stage larvae to undergo temperature-induced exsheathing and/or metamorphosis in the absence of a host.

In summary, the quality of xWORM assays is influenced by numerous interrelated parameters, including media type, media concentration, in-well parasite density, and assay duration. Furthermore, the effect of these parameters was found to vary between closely related helminth species at the same larval stage. This reveals that there are species-specific enviro-physiological differences in hookworms that can impact the conditions necessary for achieving high-quality xWORM assays. We anticipate that these effects are likely relevant to other helminth species and life stages as well. Moreover, we have emphasised the importance of several other critical assay parameters, including pH, temperature, and nutrient and oxygen availability, which should be considered in future works for their potential impact on larval viability and assay quality. This study highlights the need for a dedicated optimization phase and a deeper understanding of the conditions under which xWORM assays are conducted, specifically their effect on parasite physiology and behavior. By addressing these factors, researchers can ensure the generation of accurate and reliable results, ultimately contributing to more effective research in the future.

## Data availability statement

The raw data supporting the conclusions of this article will be made available by the authors, without undue reservation. The robust collection of Motility Index (MI) and Strictly Standardised Mean Difference Prime (SSMD’) figures generated for all conditions investigated in this study is publicly accessible through the following link: https://doi.org/10.5281/zenodo.7927398.

## Ethics statement

The studies involving human participants were reviewed and approved by James Cook University Human Research Ethics Committee. The patients/participants provided their written informed consent to participate in this study. The animal study was reviewed and approved by James Cook University Animal Research Ethics Committee.

## Author contributions

DL-B, MS, and JS contributed to the conception and design of this study. DL-B, MS, and LB were involved in data collection. DL-B, JS, MS, and AL aided in data analysis and presentation. DL-B wrote the original draft. All authors contributed to the final article and approved the submitted version.
