# Supplementary material for: Optimizing the xWORM assay for monitoring hookworm larvae motility
Source: Front Parasitol. 2023 Jun 19;2:1189872. doi: 10.3389/fpara.2023.1189872 (PMC11731822; doi:10.3389/fpara.2023.1189872)
Supplement: Supplementary file 1 [file DataSheet_1.docx]

Supplementary Material

# Supplementary Data 1

To illustrate our data processing method, here we provide an example of what a typical cell index (CI) scatter would look like between favourable conditions (500 *N. brasiliensis* L3 in 25% PBS), detrimental conditions (1000 *N. brasiliensis* L3 in 100% PBS), and control groups (100% PBS media only, and 500 heat-killed L3 *N. brasiliensis* in 100% PBS) over time (Supplementary Figure 1). CI amplitude (i.e., parasite motility) varied between favourable conditions, detrimental conditions, and control groups. The CI amplitude of the media-only control was stable with low variation between the data points over the 24 hours of data collection. Heat-killed parasite controls showed a slightly higher amplitude but were still comparatively close to the CI output of media-only controls. However, detrimental conditions showed a high variance (i.e, motility) for the first three hours, which rapidly declined, and by nine hours was approaching the signal of the heat-killed parasites control, suggesting that these conditions may not be ideal for larvae longevity. Conversely, while favourable conditions exhibited a lower signal initially, the amplitude of the signal remained stable over the 24-hour test period. This stability is key to enhancing the sensitivity of assays aiming to screen novel compounds for anthelmintic activity.

**Supplementary Figure 1.** **RTCA unit differentiates between live and dead parasites, as well as optimal and detrimental assay conditions using the Cell Index readout.** An example of the cell index output generated by *Nippostrongylus brasiliensis* (rodent hookworm) L3 in favourable assay conditions (**x**, 500 L3 *N. brasiliensis* in 25% PBS) versus detrimental assay conditions (**x**, 1000 *N. brasiliensis* L3 in 100% PBS) as well as heat-killed parasites (**x**, 500 heat-killed *N. brasiliensis* L3 in 100% PBS) and media only (**x**, 100% PBS) control groups. The vertical spread (amplitude) of the cell index at each time point represents changes in electrical resistance which can be used to monitor the motility of the larvae in real time.

# Supplementary Data 2

Here we show the previously presented CI data for *N. brasiliensis* L3 in favourable conditions, detrimental conditions, and positive controls after conversion to MI and blank (i.e., media-only control) adjustment (Supplementary Figure 2). The heat-killed parasites control group maintained a stable motility close to zero, indicating total mortality, with minimal variation between replicates over 24-hours. Additionally, the same trends emerged where favourable conditions resulted in a stable motility with low variation between replicates over 24 hours, and detrimental conditions initially induced hypermotility with high variation between replicates which rapidly declined towards the heat-killed parasites control group over ~15 hours.

**Supplementary Figure 2. Motility Index (MI) transformations simplify the visualisation and interpretation of raw Cell Index (CI) data generated by the RTCA unit.** An example of mean blank-adjusted MI for infective (L3) rodent hookworm *Nippostrongylus brasiliensis* larvae over time. MI traces are of *N. brasiliensis* L3 in favourable assay conditions (●, 500 *N. brasiliensis* L3 in 25% PBS), and detrimental assay conditions (●, 1000 *N. brasiliensis* L3 in 100% PBS), as well as heat-killed parasite controls (●, 500 heat-killed *N. brasiliensis* L3 in 100% PBS). Error bars are 95% CI.
